# Species-Specific Hepatic Uptake of [^64^Cu]Cu-EOB-NOTA, A Newly Designed Hepatospecific PET Agent

**DOI:** 10.1007/s11307-025-02009-0

**Published:** 2025-04-30

**Authors:** Jinda Fan, Bijja Janaki Ramulu, Christiane L. Mallett, Legend E. Kenney, Nathan Kauffman, Tapas Bhattacharyya, Maryam Sabbaghan, Satyendra Singh, Kurt R. Zinn, Erik M. Shapiro

**Affiliations:** 1 Department of Radiology, Michigan State University, 846 Service Rd, East Lansing, MI 48824 USA; 2 Department of Chemistry, Michigan State University, East Lansing, MI USA; 3 Department of Physiology, Michigan State University, East Lansing, MI USA; 4 Department of Chemical Engineering and Material Science, Michigan State University, East Lansing, MI USA; 5 Department of Biomedical Engineering, Michigan State University, East Lansing, MI USA; 6 Institute for Quantitative Health Science and Engineering, Michigan State University, East Lansing, MI USA; 7 Chemistry Department, Faculty of Science, Shahid Rajaee Teacher Training University, Tehran, Iran; 8 Department of Small Animal Clinical Sciences, Michigan State University, East Lansing, MI USA

**Keywords:** Liver, PET imaging, MRI, Copper, Mouse

## Abstract

**Purpose:**

Measuring hepatic flux rates of transportable substrates has the potential for assessing liver function. PET imaging of a PET-enabled substrate may provide a more straightforward measurement of time-dependent substrate concentration through the liver than MRI using an MRI contrast agent. Here we synthesized and evaluated the hepatobiliary transport of a new hepatospecific PET agent designed for stable Cu^2+^ chelation and transport by hepatic OATPs, [^64^Cu]Cu-EOB-NOTA.

**Procedures:**

EOB-NOTA was synthesized, its two enantiomers separated by chiral HPLC, and individually radiolabeled with [^64^Cu]Cu^2+^. Cocktails of each enantiomer of [^64^Cu]Cu-EOB-NOTA and Gd-EOB-DTPA were formulated for simultaneous PET/MRI imaging of hepatic flux by PET and MRI. Two mouse models were evaluated: wild-type mice and mice expressing only human hepatic OATPs.

**Results:**

In wild-type mice, [^64^Cu]Cu-EOB-NOTA hepatic influx and efflux was high, but slower compared to Gd-EOB-DTPA. Neither enantiomer of [^64^Cu]Cu-EOB-NOTA exhibited detectable transport into the liver in mice expressing human OATPs. This was validated by waste clearance studies and *in vitro* uptake assays in cells engineered to express rodent and human OATPs.

**Conclusion:**

[^64^Cu]Cu-EOB-NOTA exhibited no detectable hepatic uptake by transgenic mice expressing human hepatic transporters. This finding was surprising given the efficient transport of the structurally similar metal chelate Gd-EOB-DTPA, and underscores challenges in the design of imaging molecular probes, including poor predictability for hepatic transport, and the value of validating new agents in mice expressing human hepatic transporters.

## Introduction

One promising technique for early detection and accurate staging of chronic liver diseases (**CLD**) is to measure subtle deficiencies in liver function such as hepatobiliary clearance of pharmaceuticals [1]. Diagnostic imaging can potentially be used to non-invasively quantify the hepatic flux of an imageable pharmaceutical that exhibits hepatobiliary clearance. The analysis of this data uses parametric estimation techniques and physiologically based pharmacokinetic (**PBPK**) models to evaluate important liver functional parameters such as k_influx_ – the influx rate of this molecule into hepatocytes via organic anion transport polypeptides (**OATPs**), and k_efflux_ – the efflux rate of this molecule from hepatocytes to bile via the multidrug resistance-associated protein 2 (**MRP2**) transporter[1–6]. Accurate and reproducible estimation of k_influx_ and k_efflux_ is important, as subtle changes in these rates may reflect early abnormalities in transporter expression in CLD such as fibrosis, cirrhosis, nonalcoholic fatty liver disease (NAFLD), and diabetes, and may enable early disease detection.

The key inputs to these PBPK models are the dynamic hepatic and vascular concentrations of the imageable molecule. Dynamic contrast enhanced-MRI (**DCE-MRI**) with the clinically-approved hepato-specific MRI contrast agent Gd-EOB-DTPA, has been investigated for this purpose [1–4]. In this procedure, Gd-EOB-DTPA is injected and dynamic T_1_-weighted MRI is acquired for several minutes to 1 h. The Gd-EOB-DTPA in the liver and blood causes MRI signal intensity enhancement, but PBPK models require time-dependent contrast agent concentration, and so MRI signal enhancement needs to be converted to contrast agent concentration. While the mathematics of this conversion is straightforward, in practice this calculation is difficult. Various parameters are often assumed and/or there are uncertainties about tissue and blood T_1_ values, contrast agent relaxivity, and MRI acquisition error sources (RF inhomogeneity, in flow effects, etc.) [7]. Taken together, the propagation of these errors results in a poor correlation between the hepatic contrast agent concentrations calculated by DCE-MRI with actual contrast agent concentrations measured in liver biopsies by analytical chemistry techniques [4]. This results in poor estimation of liver function and the inability of DCE-MRI to detect early liver disease or discriminate stages of CLD.

Unlike MRI contrast agents, PET tracer concentrations can in principle be measured directly from images, and PET is devoid of those imaging-related artifacts that hamper MRI as described above. Because of this, we hypothesize that dynamic PET imaging can measure the concentration flux of hepato-specific imaging probes in the liver and in the vasculature more accurately than MRI. This would enable more exact measurements of hepatic k_influx_ and k_efflux_, and better detection of early disease and disease stage. Accurate determination of vascular input functions to the liver has been accomplished with ^18^F PET probes [8], but chemically modifying Gd-EOB-DTPA with an ^18^F has not been reported. Since it is vitally important to include an accurately measured vascular input function into PBPK models, we hypothesize that by using a ^64^Cu PET radioisotope, with a theoretical image resolution of 0.7 mm, similar to that of ^18^F [9], we will be able to obtain an accurate image-derived vascular input function by confining the PET signal within small blood vessels that feed the liver. Thus, our goals here were to synthesize a ^64^Cu-based PET probe with high hepato-specificity and rapid hepatic flux and to evaluate its hepatic flux in mice.

**Fig. 1:**
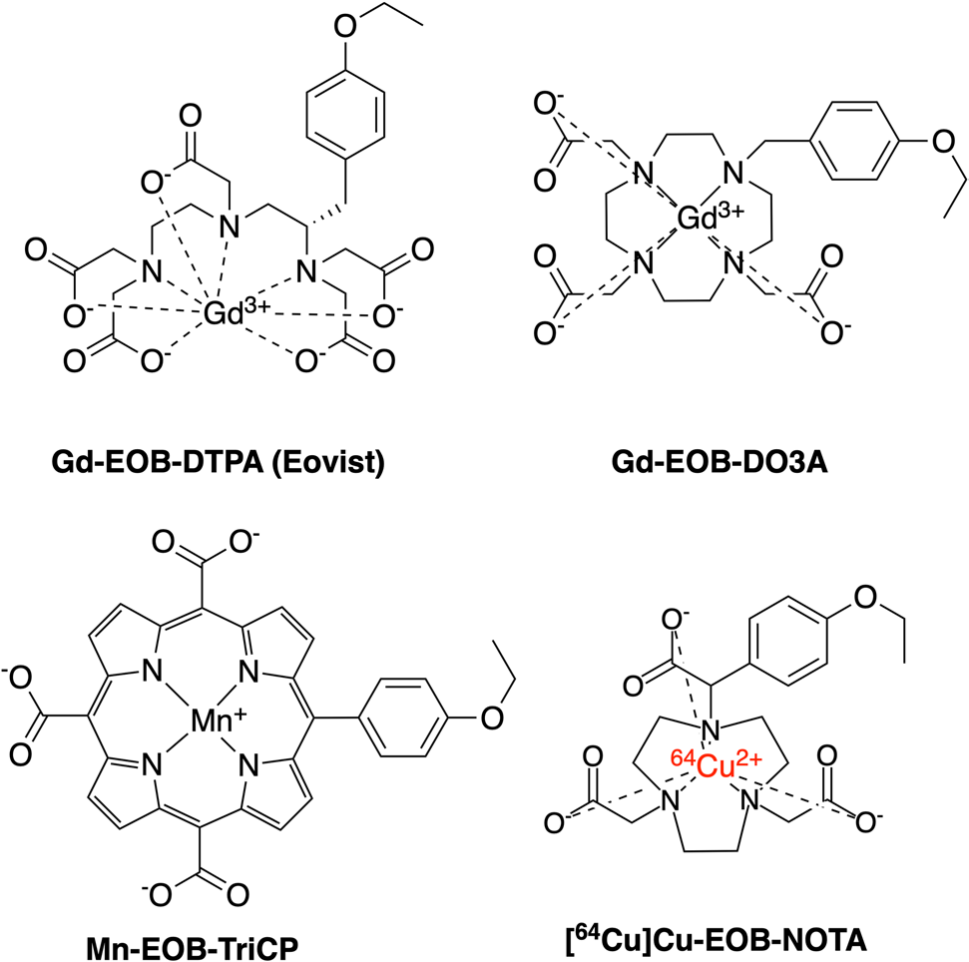
Structures of Gd-EOB-DTPA, Gd-EOB-DO3A, Mn-EOB-TriCP and [^64^Cu]Cu-EOB-NOTA

In designing a ^64^Cu-based PET probe with high hepato-specificity and rapid hepatic flux we considered that the hydrophobic ethoxybenzene (**EOB**) moiety, when appended to DTPA as EOB-DTPA, is sufficient to transform Gd-DTPA, which exhibits no transport by hepatic OATPs, into the clinical transportable substance, Gd-EOB-DTPA [10]. This same principle has been reported for Gd-DO3A, where the addition of EOB to the otherwise non-transportable Gd-DO3A to create Gd-EOB-DO3A engenders transport activity [11]. Further, the addition of EOB to the otherwise non-transportable Mn-tri(carboxyl)-porphyrin to create Mn-EOB-TriCP also engenders OATP transport [12]. As such, we hypothesized that adding EOB to otherwise non-transportable [^64^Cu]Cu-NOTA to generate [^64^Cu]Cu-EOB-NOTA would impart hepatic transport capabilities. Figure 1 shows the structures for these four EOB-modified agents.

Hepatic OATPs are responsible for the transport of EOB-like hepatospecific contrast agents into liver, but there is species dependent uptake of chemicals. For example, rodents efficiently transport both Gd-BOPTA and Gd-EOB-DTPA through OATP1B2 (and OATP1A1) [13, 14], but humans efficiently transport only Gd-EOB-DTPA and very poorly transport Gd-BOPTA through OATP1B1 and OATP1B3 [15]. To maximize the translational information obtained in this study, we evaluated this new complex in both wild-type mice which express OATP1B2 and OATP1A1 and in chimeric OATP1B1/1B3 knock-in mice (OATP1A/1B cluster knockout) with human OATP1B1 and OATP1B3 expressed in the liver [16]. We refer to these mice herein as liver-humanized mice. In liver-humanized mice, OATP expression is driven from the apolipoprotein E (APOE) promoter, restricting expression to the liver. These liver-humanized mice accurately recapitulate the clearance of imageable molecular probes from the blood following IV injection found in humans while wild-type mice do not [16]. Thus, the information we obtained herein can be more easily be extrapolated to humans.

## Materials and Methods

### Synthesis and Chemical Analysis

The NOTA-like starting material was purchased from Macrocyclics (Plano, TX). HPLC-grade acetonitrile and all other chemical reagents were purchased from Sigma Aldrich (St. Louis, MO). NMR spectra were recorded on a Bruker (Karlsruhe, Germany) NMR spectrometer (500 and 600 MHz for ^1^H, 126 and 150 MHz for ^13^C). ^1^H-NMR chemical shifts were measured relative to tetramethylsilane (TMS). The organic compounds were purified using column chromatography on silica gel. The final compounds (tricarboxylic acid) were purified using an Agilent 1220 HPLC system (Santa Clara, CA). The solvent systems were used: solvent A (0.1% TFA in water) and solvent B (0.1% TFA in acetonitrile). The flow rates were 1 mL/min for analytical HPLC (4.6 mm diameter column) and 4 mL/min for semipreparative HPLC (9.2 mm diameter column) at the indicated method. Electro-spray ionization mass spectra (ESI–MS) were acquired using a Waters (Milford, MA) ESI ion trap spectrometer using positive and negative ion detection.

### Synthetic Procedures

*Tert*-butyl 2-(4-ethoxyphenyl)acetate (**2**) To a solution of 2-(4-ethoxyphenyl)acetic acid (2 g), in dichloromethane (DCM) added ^t^BuOH (1.2 equiv) and DMAP was dissolved at 0 ºC. The reaction was allowed to come to room temperature and stirred overnight. After completion of reaction monitored with TLC, the reaction mixture was filtered, and the filtrate was workup with H_2_O and extracted with DCM solvent. The organic layer (DCM) was evaporated using vacuum and purified with column chromatography using silica gel. The product yield 92%. ^1^H NMR (500 MHz, CDCl_3_) *δ* 7.17 (d, *J* = 8.6 Hz, 2H), 6.84 (d, *J* = 8.7 Hz, 2H), 4.01 (q, *J* = 7.0 Hz, 2H), 3.45 (s, 2H), 1.43 (s, 9H), 1.40 (t, *J* = 7.0 Hz, 3H). ^13^C NMR (126 MHz, CDCl_3_) *δ* 171.32, 157.85, 130.17, 126.66, 114.43, 80.63, 63.38, 41.73, 28.04, 14.86.

*tert*-butyl 2-bromo- 2-(4-ethoxyphenyl)acetate (**3**) To a solution of** 2** in CCl_4_ (20 mL) added NBS (1.5 equiv.) and AIBN (0.1 equiv.) the reaction was stirred at 80 ºC for 3–4 h. After completion of reaction monitored with TLC, the reaction mixture transferred into room temperature and quenched with H_2_O. After workup with H_2_O and DCM solvent the organic layer was evaporated using rotavapor and purified with column chromatography using silica gel. ^1^H NMR (500 MHz, CDCl_3_) *δ* 7.46 (d, *J* = 8.8 Hz, 2H), 6.86 (d, *J* = 8.8 Hz, 2H), 5.24 (s, 1H), 4.03 (q, *J* = 7.0 Hz, 2H), 1.46 (s, 9H), 1.42 (t, *J* = 6.8 Hz, 3H). ^13^C NMR (126 MHz, CDCl_3_) *δ* 167.39, 159.50, 130.03, 129.25, 128.06, 114.59, 82.90, 63.51, 48.40, 27.74, 14.77.

di-*tert*-butyl 2,2'-(7-(2-(*tert*-butoxy)-1-(4-ethoxyphenyl)-2-oxoethyl)-1,4,7-triazonane-1,4-diyl)diacetate (**5**) To a solution of di-tert-butyl 2,2'-(1,4,7-triazonane-1,4-diyl)diacetate (**4**) and **3** in CH_3_CN (20 mL) solvent added K_2_CO_3_ (5 equiv.). The reaction was allowed at 60 ºC for 3–5 h. after completion of reaction monitored with TLC the solvent was evaporated using rotavapor and workup done with ethyl acetate and H_2_O. The organic layer was concentrated using rotavapor. The yellow liquid product was obtained with a yield of 62%. ^1^H NMR (500 MHz, CDCl_3_) *δ* 7.29 (d, *J* = 8.7 Hz, 2H), 6.82 (d, *J* = 8.7 Hz, 2H), 4.35 (s, 1H), 4.01 (q, *J* = 7.0 Hz, 2H), 3.27 (d, *J* = 3.2 Hz, 4H), 3.01 (d, *J* = 11.4 Hz, 2H), 2.89–2.74 (m, 10H), 1.44 (s, 9H), 1.43 (s, 18H), 1.40 (t, *J* = 7.0 Hz, 3H). ^13^C NMR (126 MHz, CDCl_3_) *δ* 172.41, 171.51, 158.29, 130.07, 130.00, 114.07, 81.00, 80.59, 72.24, 63.31, 59.50, 55.38, 55.03, 53.40, 28.19, 28.14, 14.85. HRMS calculated for C_32_H_54_N_3_O_7_, 592.3962 [M + H +], found: 592.4023 [M + H +]. Enantiomeric resolution of **5a** and **5b**, A CHIRALPAK IC column (9.2 × 250 mm, 5 μ) was used for the resolution, solvent was hexane/isopropanol (96/4), with a flow rate of 4 mL/min. **5a** had a retention time of 13.4 min and **5b** had a retention time of 18.1 min, respectively. The collected compounds were dried under vacuum with rotavapor. The optical rotation of **5a** is [a] _D_^20^ = + 0.8 deg·mL·g^−1^·dm^−1^ and that of **5b** is [a] _D_^20^ =—0.7 deg·mL·g^−1^·dm^−1^.

2,2'-(7-(carboxy(4-ethoxyphenyl)methyl)-1,4,7-triazonane-1,4-diyl)diacetic acid (**6**) To a solution of **5**, **5a** or **5b**, added 4 M HCl in 1,4-dioxane (10 equiv). The reaction was allowed at room temperature for 3–5 h, monitored with HPLC. After completion of reaction, the product was purified using a C18 HPLC column (mobile phase water and acetonitrile). The compounds **6**, **6a** and **6b** were concentrated using a lyophilizer. Yield was 30% ± 15%. ^1^H NMR (600 MHz, DMSO) *δ* 7.29 (d, *J* = 8.7 Hz, 2H), 6.90 (d, *J* = 8.7 Hz, 2H), 4.79 (s, 1H), 4.08–3.94 (m, 4H), 3.88 (d, *J* = 17.7 Hz, 2H), 3.75 (s, 3H), 3.42 (d, *J* = 6.0 Hz, 2H), 3.10 (td, *J* = 13.9, 6.7 Hz, 3H), 2.98 (dt, *J* = 31.0, 6.8 Hz, 5H), 2.84–2.74 (m, 3H), 1.32 (t, *J* = 6.9 Hz, 3H). ^13^C NMR (150 MHz, DMSO) *δ* 173.6, 171.1, 170.7, 158.7, 130.9, 128.1, 117.9, 116.0, 114.7, 67.4, 63.5, 55.1, 54.4, 51.0, 50.5, 49.3, 48.5, 47.3, 15.0. HRMS calculated for C_20_H_30_N_3_O_7_, 424.2078, [M + H^+^], found: 424.2133 [M + H^+^].

### Radiochemistry

[^64^Cu]Cu^2+^ (130–160 MBq) was provided in 0.1 M HCl. After HCl was evaporated at 120 ºC, [^64^Cu]Cu^2+^ was dissolved in 50 µL of 1.0 M acetate buffer (pH 5.0–5.5). Then **6a** or **6b** (5 µg (0.011 µmol)/5 µL of water) was added and incubated for 30 min at 37 ºC with constant shaking on an Eppendorf Thermomixer. Labeling yield was found to be 92% and 94% for **6a** and **6b,** respectively, determined by radiochemical thin layer chromatography (radio-TLC). TLC analyses were performed with silica plates (Silica gel 60 mesh, Merck, Darmstadt, Germany) with 1 M aqueous ammonium acetate – methanol (1:1) as the developing solvent. **[**^**64**^**Cu]Cu-6a** and **[**^**64**^**Cu]Cu-6b** were purified using PD- 10 columns with phosphate-buffered saline (PBS) as the mobile phase. The radioactive fractions containing **[**^**64**^**Cu]Cu-6a** and **[**^**64**^**Cu]Cu-6b** were collected and passed through a 0.2 µm syringe filter for *in vivo* experiments. Higher than 90% yield (decay-corrected) was achieved after purification. The radiochemical purities of **[**^**64**^**Cu]Cu-6a** and **[**^**64**^**Cu]Cu-6b** were determined by RP-HPLC and TLC being higher than 99%. Reversed-phase HPLC (RPHPLC) analyses were performed with a C18 column eluted with a linear gradient of a 10–90% mixture of acetonitrile and 0.1% aqueous TFA. Specific activity of the **[**^**64**^**Cu]Cu-6a** and **[**^**64**^**Cu]Cu-6b** were 13.2 ± 4.6 GBq/µmol (n = 4). The stability of **[**^**64**^**Cu]Cu-6a** and **[**^**64**^**Cu]Cu-6b** was assessed by sitting the final dose in PBS (pH 7.4) at room temperature for 1, 3, 6 and 18 h, the samples were analyzed by HPLC to measure the amount of free ^64^Cu dissociated from **[**^**64**^**Cu]Cu-6a** and **[**^**64**^**Cu]Cu-6b.**

### *In Vivo* PET/MRI

Images were acquired on a Bruker BioSpec 70/30 7T MRI with a PET insert for simultaneous PET/MRI, using Paravision 360v3.2 and v3.5. Mice were either wild-type (WT) mice (FVB, n = 2, ages 40 weeks, BALB/c, n = 4, ages 60 weeks, all female), or liver humanized mice (n = 6, ages 22–40 weeks, 4 female, 2 male) (used under license from Taconic). DCE-MRI was acquired using a 40 mm volume transmit/receive coil using a retrospectively respiration-gated sequence, IgFLASH with: TR/TE 80/2.1 ms, 6 coronal slices with 0.25 × 0.2x1 mm resolution, 6 (WT) or 8 oversamples (liver humanized), flip angle 20°, temporal resolution 1–1.25 min. PET images were corrected for decay and scatter and reconstructed using a maximum-a-posteriori (MAP) algorithm at 0.5 mm voxel size, with 12, 5-min bins. We injected each mouse with 0.025 mmol/kg of Gd-EOB-DTPA mixed with ~ 75 ± 31 µCi (0.094 ± 0.039 µg) of [^64^Cu]Cu-EOB-NOTA through a tail vein catheter after 5–6 min of baseline scans. To demonstrate the difference between transporter-mediated uptake and blood-pool uptake, one liver humanized mouse was scanned in a 72 mm transmit/receive coil with a gradient echo T_1_ weighted FLASH sequence to allow for whole body MRI, with: TR/TE 12.5/2 ms, 1 coronal slice with 0.5 × 0.5x2 mm voxel size, 30° flip angle, 60 averages, 1 min 15 s temporal resolution. Additionally, one liver humanized mouse was scanned in the 40 mm volume coil as described above, with co-injection of [^64^Cu]Cu-EOB-NOTA (A) and 0.025 mmol/kg Gd-DO3A-butrol (Gadobutrol), a clinically approved blood pool contrast agent.

### Image Analysis

All images were segmented in PMOD 4.2. For DCE-MRI images, circular ROIs with 1.5–2 mm radius were placed in the liver and the signal over time was measured. The data was then exported to Microsoft Excel where the percent enhancement was calculated (S(t)-S(0–5))/S(0–5)* 100. The total injected dose was measured from the whole-body PET image as the maximum total activity in the first 3 frames of the dynamic scan. ROIs from the DCE-MRI measurements were transferred to the PET images co-registered to those scans to measure the TAC, which was then expressed as % injected dose/mL.

### Analysis of Waste Clearance

4 mice (FVB enantiomer A or B, liver humanized mice enantiomer A or B) were singly housed in metabolic cages (Tecniplast, Italy) immediately after their PET/MRI scan. Feces and urine were collected after ~ 20 h and activity was measured in a dose calibrator (CRC- 55 tR, Capintec).

### *In Vitro* Cellular Transport Assays

For *in vitro* transport assays, HEK293T cells stably overexpressing mouse OATP1B2 or human OATP1B3 were used. To each well of a 6-well plate, 1 × 10^4^ cells were seeded and incubated at 37 °C and 5% CO_2_ overnight, one plate for each cell line. The next day, 20 μCi of [^64^Cu]Cu-EOB-NOTA -A or -B was added to each well (N = 3 for each cell line) and incubated at 37°C and 5% CO_2_ for 2 h at gentle shaking. Next, the cell culture supernatant was removed, and cells were washed thrice with PBS. To lyse the cell monolayer, 1 mL of 1 M NaOH was added and incubated for 10 min rocking at 700 rpm. The whole cell lysates were collected, and activity was counted on a Gamma Counter (Wizard2, Perkin Elmer). Protein concentration was measured using a NanoDrop spectrophotometer (Thermo Scientific) at an A280 setting (280 nm wavelength). The total activity for each cell lysate was normalized with the protein concentration to calculate activity per microgram of protein.

## Results and Discussion

### Synthesis and Characterization of [^64^Cu]Cu-EOB-NOTA

**Fig. 2:**
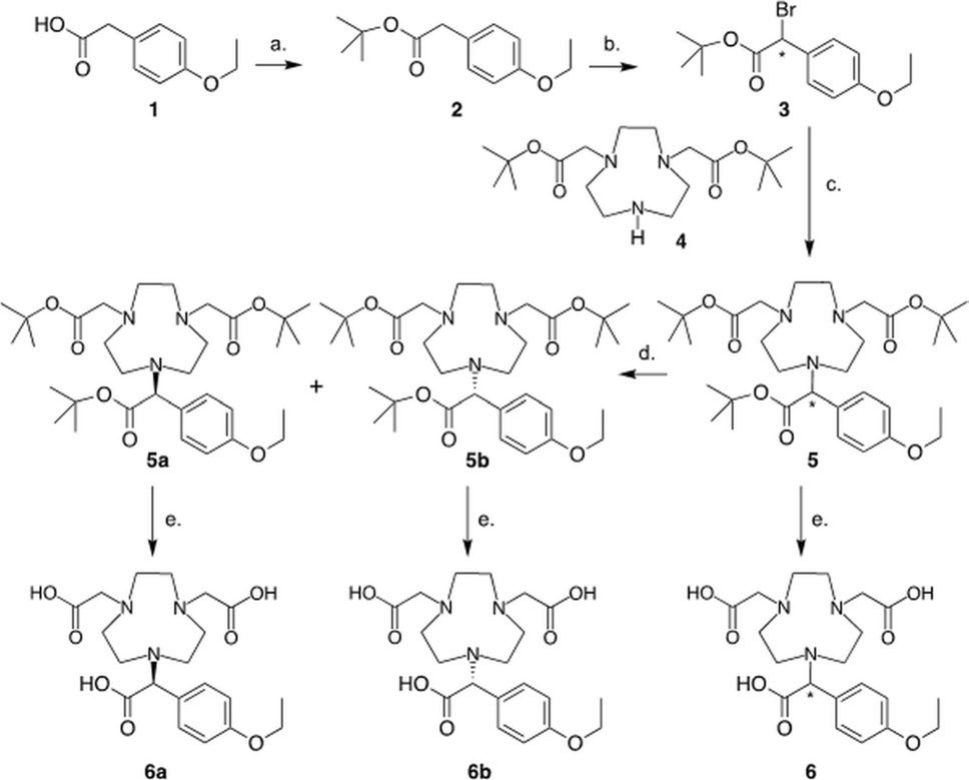
Synthesis of precursor molecules, compounds **6a** and **6b**. Reaction conditions: a. ^*t*^BuOH, DCC, DMAP, DCM, 0 ºC to r.t., 12 h, 92%. b. NBS, AIBN, CCl_4_, 80 ºC, 3–4 h, 75%. c. K_2_CO_3_, acetonitrile, 60 ºC, 3 h, 62%. d. Chiral HPLC, Chiral IC column, hexane:isopropanol (96:4). e. 4 M HCl in 1,4-dioxne, r.t., 42%

We chose ^64^Cu as the radionuclide as it has the highest resolution of the PET radiometals, an important feature to enable us to eventually measure image derived vascular input function. 1,4,7-triazacyclononane-1,4,7-triacetic acid (NOTA) is a macrocyclic, multidentate chelator with the capacity to form stable complexes with Cu^2+^. NOTA tolerates modifications and several NOTA-based bifunctional chelators bearing reactive functional groups have been developed, possessing either carboxyl, amino or isothiocyanate groups for conjugation reactions with various biologically active molecules. The synthesis of EOB-NOTA ester (**5**) was accomplished as presented in Fig. 2. From commercial 2-(4-ethoxyphenyl)acetic acid (**1**), the carboxylic acid was first protected with *t*-butanol to form *tert*-butyl-2-(4-ethoxyphenyl)acetate (**2**). Then a bromine atom was introduced to the benzylic position through a radical bromination reaction, obtained *tert*-butyl 2-bromo- 2-(4-ethoxyphenyl)acetate **3**. The EOB-NOTA Ester (5) was prepared from the coupling of di-tert-butyl-2,2'-(1,4,7-triazonane- 1,4-diyl)diacetate (**4**) and tert-butyl-2-bromo- 2-(4-ethoxyphenyl)acetate (**3**). Up to EOB-NOTA ester (**5**), all reactions were achieved in great to good yield. The hydrolysis of *tert*-butanol ester groups was problematic and several methods were attempted (see Table 1) for the deprotection *tert*-butyl group. TFA treatment was the most commonly used method for the removal of *t*-butanol ester groups. However, in the presence of TFA (started from 1.5 to > 60 equivalents) every time the result was showing either partially deprotected or fully decomposed product. A fully deprotected product could not be obtained. This was attributed to the fact that in the presence of TFA protected PMB related products are not stable [17]. Next, we attempted deprotection in presence of Yb(OTf)_3_ [18]_._ In mass spectrometry, we observed fully deprotected product. The possibility of forming Yb^3+^-NOTA excluded this method for the synthesis of final product. This method provided some fully deprotected product that we could use as reference standard to develop HPLC method for the monitoring of the deprotection process. Then, two other methods were tried. One reaction was conducted in presence of Iodine in mixture of H_2_O and CH_3_CN solvent at 80 °C, after confirmation fully deprotected product with mass spectrometry further purified with HPLC [19]. However, the product was obtained in low yield. The other method was using KSF clay [20]. Fully deprotected product was confirmed by mass spectrometry, but the product was obtained in low yield after HPLC purification, probably because of the physical loss caused by the adsorption of the polarized product to the KSF clay. Finally, the method of 4 M HCl in 1,4-dioxane solvent worked for us [21]. We were able to obtain the product compound **6** in moderate yield (Table 1).

**Table 1:** Deprotection of *t*-butanol esters on compound 5

Entry	Method	Temp (^o^C)	Yield %
1	TFA/DCM	r.t	-
2	LiOH/H_2_O: THF	r.t.− 80	-
3	Yb(OTf)_3_	65	N.D
4	I_2_/H_2_O:CH_3_CN	r.t	< 5
5	KSF Clay/CH_3_CN	80	< 5
6	4 M HCl in 1,4-Dioxane	r.t	30% ± 15%

**Fig. 3:**
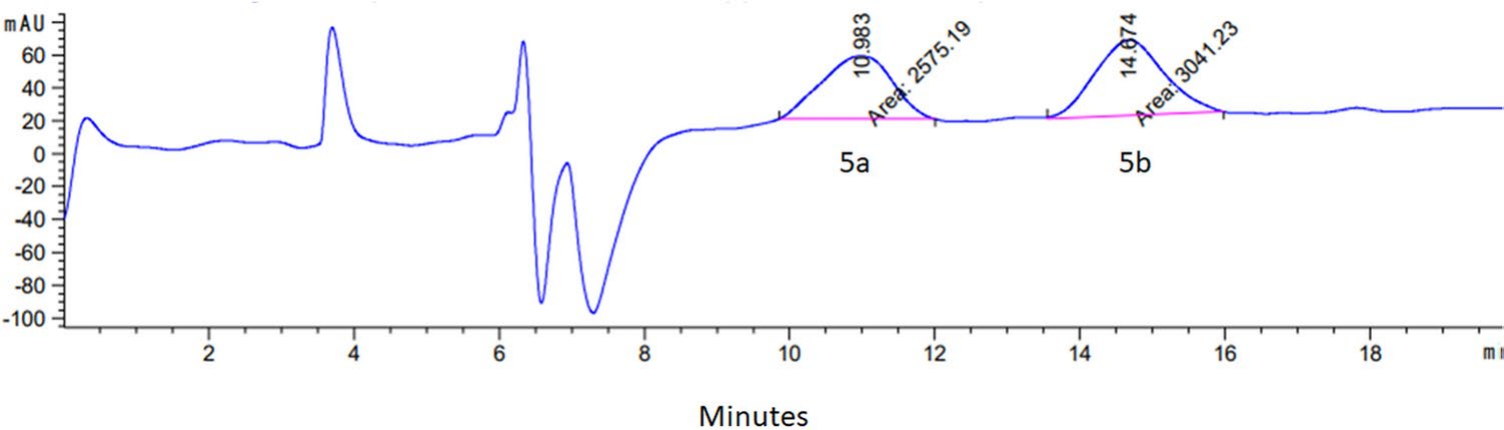
Chiral HPLC showing resolved enantiomers 5a and 5b

The EOB-NOTA compound contains one chiral center. Chiral HPLC method was developed and the two enantiomers were resolved. Initially, we tried with a Chiralcell OD-H column to resolve the fully deprotected product; however, we could not separate the two enantiomers with this column. The high hydrophilicity of EOB-NOTA might have reduced the separation efficiency of the chiral column. Thus, we tried to resolve the EOB-NOTA ester compound **5**. We were able to resolve the two enantiomers **5a** and **5b**, using a CHIRAL IC column (Fig. 3). The optical rotation of **5a** and **5b** are [a]_D_^20^ = + 0.8 degree, [a]_D_^20^ = − 0.7 degree, respectively.

### Radiochemistry

Compounds **6a** and **6b** were labeled with ^64^Cu radionuclide in high yield by incubation of [^64^Cu]Cu^2+^ with the precursor compounds at 37 ºC for 30 min in ammonium acetate buffer (pH 5.0–5.5) [22]. The completion of the radiolabeling was monitored by iTLC. Free ^64^Cu was < 5%. After the removal of the free ^64^Cu with a PD- 10 column. The final dose was filtered through a 0.22 µm syringe filter. The purity of the product was confirmed by a radio-HPLC of > 99%. Finally, the final dose was used for *in vitro* and *in vivo* studies.

The stability of **[**^**64**^**Cu]Cu-6a** and **[**^**64**^**Cu]Cu-6b** was assessed by sitting the final dose in PBS (pH 7.4) at room temperature for 1, 3, 6 and 18 h, the samples were analyzed by HPLC to measure the amount of free 64 Cu dissociated from **[**^**64**^**Cu]Cu-6a** and **[**^**64**^**Cu]Cu-6b**. The results confirmed that **[**^**64**^**Cu]Cu-6a** and **[**^**64**^**Cu]Cu-6b** are stable in PBS pH7.4 at room temperature.

### *In Vivo* Simultaneous PET/MRI of [^64^Cu]Cu-EOB-NOTA in Mouse Liver

**Fig. 4:**
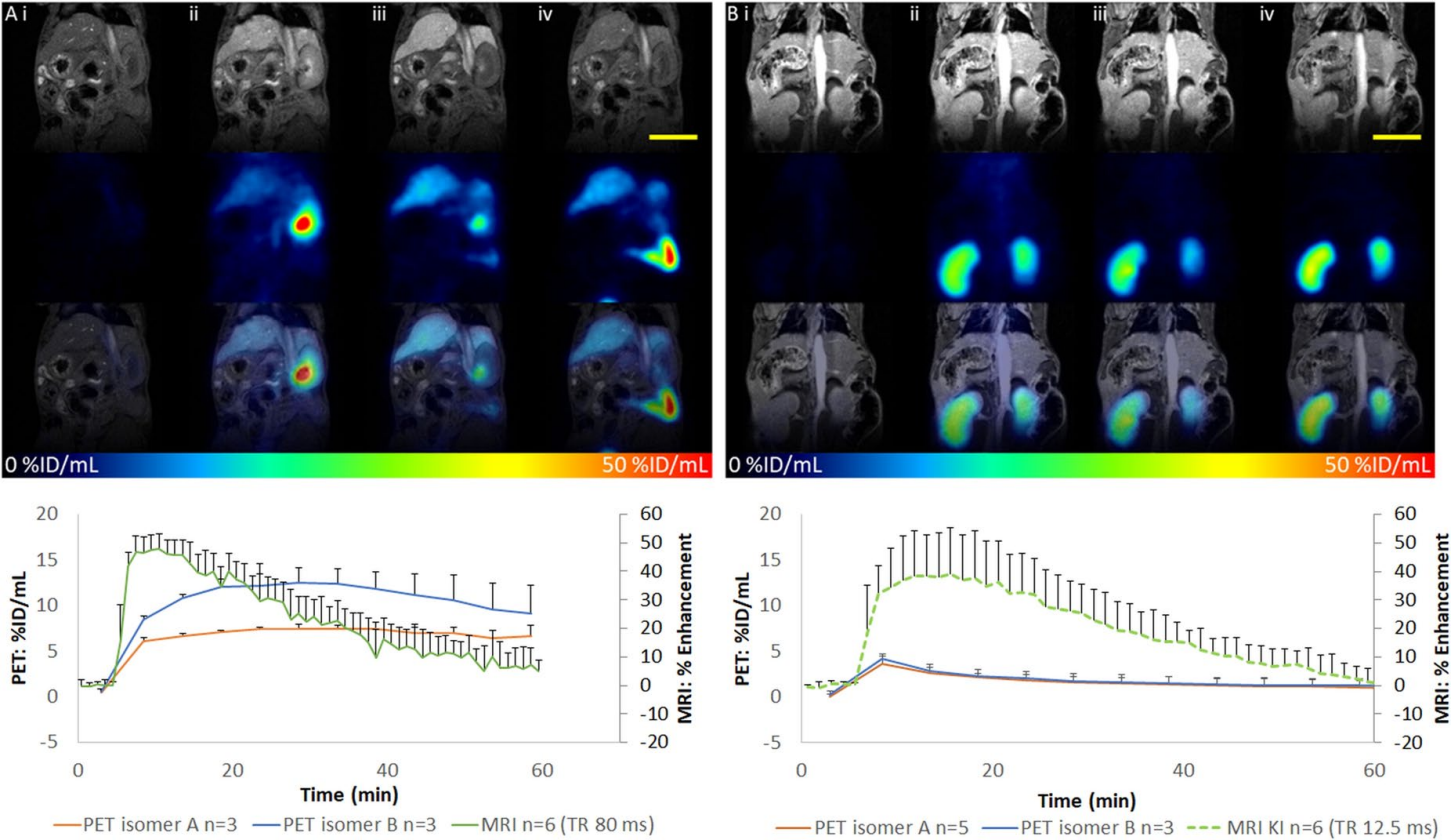
Top panels: Repressentative MRI and PET of mice after injection with Gd-EOB-DTPA and [^64^Cu]Cu-EOB-NOTA (enantiomer B). A) Wild-type mouse. B) OATP1B1/1B3 knock in mouse. i) baseline, ii) 2 min after injection, iii) 12 min after injection, iv) 60 min after injection. Top row is DCE-MRI; middle row is dynamic PET; bottom row is overlay of PET/MRI. Bottom panels: Dynamic uptake of MRI and PET agents in liver for wild-type mice and OATP1B1/1B3 knock in mice

We evaluated the hepatic flux of [^64^Cu]Cu-EOB-NOTA using simultaneous PET/MRI in 2 different mouse models; wild-type mice and liver-humanized mice. A cocktail of each enantiomer of [^64^Cu]Cu-EOB-NOTA (66 ± 9 µCi) and Gd-EOB-DTPA (0.025 mmol/kg) was mixed to enable simultaneous imaging of the hepatic flux of both agents by PET ([^64^Cu]Cu-EOB-NOTA) and MRI (Gd-EOB-DTPA). Simultaneous imaging of both PET and MRI substrates serves as both an internal control as well as a means to make direct comparisons of hepatic flux rates between molecules in identical physiological states. This cocktail was injected into mice via tail vein catheter and dynamic simultaneous PET/MRI was performed at 7.0 T for 60 min. Figure 4 shows representative images and dynamic signal traces for PET and MRI. In wild-type mice, [^64^Cu]Cu-EOB-NOTA had a relatively slow influx and efflux compared to Gd-EOB-DTPA. Peak uptake of [^64^Cu]Cu-EOB-NOTA occurred between 20–30 min post-injection while for Gd-EOB-DTPA peak uptake was ~ 7 min post-injection. Further, [^64^Cu]Cu-EOB-NOTA exhibited very slow efflux from the liver during the 60-min imaging epoch while Gd-EOB-DTPA completely cleared. This pharmacodynamic behavior of Gd-EOB-DTPA in rodents is well known and our results are in line with our published data [14, 16]. Interestingly, the two enantiomers had differential uptake despite being delivered at the same dose, with enantiomer B having ~ 60% higher peak uptake than enantiomer A. Also, it appears as though enantiomer B also exhibits faster efflux from the liver, as evidenced by the slight reduction in PET signal from ~ 35 min, though the efflux is very small and 60 min acquisition may not be long enough to fully validate this. Area under the curve was significantly higher for B than A (t-test, p < 0.05).

In experiments performed in liver humanized mice, we determined that neither enantiomer of [^64^Cu]Cu-EOB-NOTA exhibited hepatic flux consistent with hepatocellular influx and efflux, though MRI confirmed Gd-EOB-DTPA influx and efflux. The time activity curves for [^64^Cu]Cu-EOB-NOTA reflect simple wash in/wash out kinetics that is nearly identical to that seen in the heart (data not shown), which has no OATPs to transport [^64^Cu]Cu-EOB-NOTA or Gd-EOB-DTPA [23]. The lack of hepatic flux is a surprising finding given that the EOB molecular moiety is key to rendering the otherwise non-transportable Gd-DTPA a transportable substrate by human OATPs.

### Waste Clearance of [^64^Cu]Cu-EOB-NOTA in Mice

To validate the imaging findings, 4 mice (1 each of the wild type or liver humanized mice, each with 1 of the 2 enantiomers) from the *in vivo* experiments were housed for 20 h after imaging in metabolic cages, enabling the collection of urine and feces. We measured the excreted radioactivity in urine and feces to determine the relative hepatobiliary and renal clearance. In wild-type mice, hepatobiliary excretion for the two enantiomers accounted for 42%(A) and 59%(B), while in liver-humanized mice, hepatobiliary excretion accounted for 7%(A) and 12%(B). These measurements are in line with the low relative hepatic uptake of the two enantiomers of [^64^Cu]Cu-EOB-NOTA observed in the PET images, though they are data from only single animals.

### *In Vitro* Cellular Uptake of [^64^Cu]Cu-EOB-NOTA

To further validate these findings, we measured the intracellular transport of [^64^Cu]Cu-EOB-NOTA in HEK293T cells engineered to overexpress either rat OATP1B2 or human OATP1B3. Enantiomers A and B of [^64^Cu]Cu-EOB-NOTA have ~ 3.0 ± 0.6 fold higher and ~ 2.5 ± 0.4 fold higher uptake, respectively, in OATP1B2 expressing cells than OATP1B3 expressing cells (no statistical significance between the two enantiomers). These values further validate the *in vivo* PET experimental findings that show that [^64^Cu]Cu-EOB-NOTA is transported in OATP1B2-expressing wild-type mice but not by liver-humanized mice.

## Conclusions

Two enantiomers of [^64^Cu]Cu-EOB-NOTA were synthesized and assayed for their hepatic flux in wild-type mice and in liver-humanized mice. While [^64^Cu]Cu-EOB-NOTA was transported into wild-type mouse liver, with some disparity between the two enantiomers, [^64^Cu]Cu-EOB-NOTA was not transported into the human-like mouse liver, corroborated by the skewed excretion dynamics. Concomitant with this, we demonstrated that ^64^Cu-EOB-NOTA is a substrate for the rodent OATP1B2 but not human OATP1B3, explaining this surprising finding. In summary, [^64^Cu]Cu-EOB-NOTA does not appear to be a promising PET probe for assessing liver dysfunction in humans. Our study in liver-humanized mice provided valuable insights that helped reach this conclusion without necessitating human trials. This work underscores the useful role of humanized mouse models in evaluating potential new tracers, contrast agents, and, more broadly, pharmaceuticals.

## Data Availability

PET/MRI data sets will be made available to researchers upon reasonable request to EMS.
